# Malaria and Parasitic Neglected Tropical Diseases: Potential Syndemics with COVID-19?

**DOI:** 10.4269/ajtmh.20-0516

**Published:** 2020-06-01

**Authors:** Julie R. Gutman, Naomi W. Lucchi, Paul T. Cantey, Laura C. Steinhardt, Aaron M. Samuels, Mary L. Kamb, Bryan K. Kapella, Peter D. McElroy, Venkatachalam Udhayakumar, Kim A. Lindblade

**Affiliations:** 1 Malaria Branch, Division of Parasitic Diseases and Malaria, Center for Global Health, Centers for Disease Control and Prevention (CDC), Atlanta, Georgia;; 2 Parasitic Diseases Branch, Division of Parasitic Diseases and Malaria, Center for Global Health, Centers for Disease Control and Prevention (CDC), Atlanta, Georgia;; 3 U.S. President’s Malaria Initiative, Centers for Disease Control and Prevention (CDC), Atlanta, Georgia

## Abstract

The COVID-19 pandemic, caused by SARS-CoV-2, have surpassed 5 million cases globally. Current models suggest that low- and middle-income countries (LMICs) will have a similar incidence but substantially lower mortality rate than high-income countries. However, malaria and neglected tropical diseases (NTDs) are prevalent in LMICs, and coinfections are likely. Both malaria and parasitic NTDs can alter immunologic responses to other infectious agents. Malaria can induce a cytokine storm and pro-coagulant state similar to that seen in severe COVID-19. Consequently, coinfections with malaria parasites and SARS-CoV-2 could result in substantially worse outcomes than mono-infections with either pathogen, and could shift the age pattern of severe COVID-19 to younger age-groups. Enhancing surveillance platforms could provide signals that indicate whether malaria, NTDs, and COVID-19 are syndemics (synergistic epidemics). Based on the prevalence of malaria and NTDs in specific localities, efforts to characterize COVID-19 in LMICs could be expanded by adding testing for malaria and NTDs. Such additional testing would allow the determination of the rates of coinfection and comparison of severity of outcomes by infection status, greatly improving the understanding of the epidemiology of COVID-19 in LMICs and potentially helping to mitigate its impact.

## INTRODUCTION

The COVID-19 pandemic caused by SARS-CoV-2, a novel coronavirus, has now reached all corners of the world, and cases have surpassed 5 million.^1^ SARS-CoV-2 is currently spreading in low- and middle-income countries (LMICs) that experience the highest rates of malaria and neglected tropical diseases (NTDs). Neglected tropical diseases refer to a diverse group of communicable diseases caused by parasites, fungi, bacteria, and viruses that occur primarily in tropical and subtropical climates; only parasitic NTDs are considered here (Table 1). With many LMICs implementing movement restrictions or ordering their populations to stay at home to limit SARS-CoV-2 transmission, the threat to essential health services is likely to be immediate, causing delays to diagnosis and treatment for other diseases, including malaria and NTDs. During the Ebola epidemic in West Africa, there were substantial reductions in all-cause outpatient visits and patients treated with antimalarial drugs^2^; modeling the potential for similar disruptions in malaria control due to COVID-19 suggests that there could be up to an estimated 769,000 deaths due to malaria in 2020 (approximately double the number seen in 2018), mostly among children younger than 5 years.^3^ Countries working toward the elimination of malaria or NTDs may face setbacks. Less obvious, but potentially important, is the possibility of SARS-CoV-2 interacting with parasitic infections and changing the rate of severe outcomes, particularly among younger populations that have been relatively less affected by COVID-19 to date.^4^

**Table 1:** Summary of principal characteristics of COVID-19, malaria, and key neglected tropical diseases

Characteristic	COVID-19	Malaria	Soil-transmitted helminths	Schistosomiasis	Chagas
Infectious agent	SARS-CoV-2	*Plasmodium falciparum*, *Plasmodium vivax*, *Plasmodium ovale*, *Plasmodium malariae*, and *Plasmodium knowlesi*	*Ascaris lumbicoides*, *Trichuris trichiura*, and hookworm (*Ancylostoma duodenale* and *Necator americanus*)	*Schistosoma mansoni*, *S. haematobium*, and *S. japonicum*	Trypanosoma cruzi
Principal symptoms of clinical disease	Fever, cough, and shortness of breath^17,48^	Fever	*Ascaris* and hookworm: transient pneumonitis. Hookworm and *Trichuris*: abdominal pain, nausea, diarrhea, and anemia	Often asymptomatic; acute infection causes fever, cough, abdominal pain, diarrhea, hepatosplenomegaly, and eosinophilia.	Fever, edema, malaise, lymphadenopathy, hepatosplenomegaly, and chagoma (skin nodule at the inoculation site)
Severe clinical disease manifestation	Acute respiratory distress syndrome^17^	Cerebral malaria, severe anemia, and acute respiratory distress syndrome^15^	*Ascaris*: acute intestinal obstruction and peritonitis. Hookworm: anemia. *Trichuris*: colitis, anemia, growth restriction, and dysentery^73^	*S. mansoni* and *japonicum*: cirrhosis and portal hypertention. *S. hematobium*: hematuria and squamous cell carcinoma of the bladder; rarely, central nervous system lesions^15^	Chronic heart disease, dilated cardiomyopathy, megacolon, and megaesophagus
Mode of transmission	Person-to-person, primarily by respiratory droplets	Mosquito vector	*Ascaris* and *Trichuris*: ingestion of eggs. Hookworm: larvae penetrate the unprotected skin	Exposure to water containing larval forms of the parasite which penetrate the skin	Triatomine vector
Age-group most affected	Adults	Children	Children and pregnant women	School-aged children for infection and adults for severe disease	Children for infection and adults for severe disease^59^

Under the assumption that public health and social distancing measures are used to mitigate the epidemic, the modeled estimates for SARS-CoV-2 infection incidence rates for LMICs, assuming comorbidity rates for all countries similar to what was seen in Wuhan, China, are projected to be around 600 infections per 1,000 population, similar to the rate anticipated for high-income countries. However, the mortality rate for LMICs (∼2 per 1,000) is projected to be about half that of the high-income countries (∼4 per 1,000).^5^ The difference in predicted mortality rates between LMICs and high-income countries is largely due to the younger age structure in LMICs; in 2020, the median age in sub-Saharan Africa is 18.7 years, compared with 38.4 years in China.^6^

Syndemics, or synergistic epidemics, occur when two or more concurrent epidemics have a deleterious interaction,^7^ that is, when coinfections result in a worse overall outcome than for either individual infection. There are many examples of important interactions between malaria and NTDs and other infectious diseases. For example, malaria plays a role in Epstein–Barr virus (EBV) infection, leading to Burkitt’s lymphoma by contributing to B-cell proliferation and increasing EBV loads^8^; HIV-infected individuals experience a greater frequency of severe malaria and increased HIV viral load following infection with *Plasmodium falciparum*^9^; several parasite–HIV coinfections are associated with increased HIV viral load and worsened immunosuppression^10^; and schistosome infections are associated with increased transmission of HIV,^11^ whereas deworming is associated with decreased HIV viral load and improved CD4 counts among HIV-infected individuals.^12^ Biological interactions between coinfecting pathogens could involve changes in host pathology related to indirect immune effects.^12^ The interplay of coinfections hinges on several host–pathogen factors and host immunodynamics.

Low- and middle-income countries in Africa suffer the greatest burden of malaria; in 2018, there were more than 200 million cases per year, with an annual incidence of 229 per 1,000 persons.^13^ Despite substantial progress in reducing malaria mortality over the past two decades, more than 400,000 malaria deaths (> 90% in sub-Saharan Africa) were estimated to have occurred in 2018.^13^ Outside of Africa, India has the greatest burden of malaria cases, accounting for 3% of the global burden.^13^ Globally, NTDs affect more than 1 billion people, especially those living in poverty, who often lack access to clean water and adequate sanitation.^14^ Africa has a disproportionate burden of NTDs and malaria, with a significant geographical overlap.^14,15^ With rapid transmission of SARS-CoV-2, many people in LMICs, particularly in Africa, soon will be coinfected with SARS-CoV-2 and *Plasmodium* spp. or one or more NTD pathogens; cases of COVID-19 in the Africa region will soon surpass 100,000.^1^ Preexisting infection with any of these parasitic infections may lead to changes in susceptibility and/or severity of COVID-19. It is unclear whether immunomodulation caused by malaria and NTDs will be beneficial or harmful when hosts are coinfected with SARS-CoV-2, but even small changes in the risk of severe outcomes due to coinfections could result in substantial changes in the impact and epidemiology of COVID-19 in LMICs.

### SARS-CoV-2 infection.

Common symptoms of infection with SARS-CoV-2 include fever, cough, shortness of breath, chills, myalgia, headache, sore throat, and new loss of taste or smell^16^; the onset of symptoms generally occurs 4–5 days after infection, although it can be as late as 14 days,^17–19^ and not all infected people develop symptoms.^20–22^ Approximately a week after the development of symptoms, some patients experience an acute worsening, with a pronounced systemic increase of inflammatory mediators and cytokines.^23^ The severe systemic inflammatory response, referred to as a “cytokine storm,” is characterized by markedly increased levels of interleukins (IL) and tumor necrosis factor (TNF)-alpha, and is associated with the development of acute respiratory distress syndrome (ARDS).^24^ Among 72,314 cases reported from China, 14% were rated as severe and 5% were critical (respiratory failure, septic shock, and multiple organ dysfunction or failure).^17^ Case fatality ratios (CFRs) ranged from 2.3% to 7.2%, with higher CFRs among older adults (8.0–12.8% among those aged 70–79 years and 14.8–20.2% among those 80 years and older, versus ≤ 0.4% among those younger than 50 years).^17,25^ Hypertension, diabetes, cardiovascular disease, preexisting respiratory disease, and obesity were common comorbidities^26,27^; in a meta-analysis of 1,576 patients in China, all but diabetes and obesity were associated with increased risk of severe disease.^27^

### Potential *Plasmodium* spp.–SARS-CoV-2 interactions.

Of the five parasitic species that cause malaria in humans (Table 1), *P. falciparum* accounts for most morbidity and mortality, followed by *Plasmodium vivax*.^13,15^ Clinical illness arises from asexual parasite replication within erythrocytes. Infected erythrocytes lyse and release merozoites into the circulation, causing activation of the immune system and leading to the release of pro-inflammatory cytokines including TNF-alpha, interferon-gamma, IL-6, and IL-12.^28^ This cascade of cytokines leads to symptoms of uncomplicated malaria, including periodic fever, which, if left untreated, can progress to severe disease. Severe disease manifests as severe anemia, respiratory failure, cerebral malaria, acidosis, and renal failure. Children and infants are at greatest risk for severe malaria; 67% of malarial deaths are estimated to occur among African children younger than 5 years.^13^

As with COVID-19, cellular immune responses in malaria involving the cytokine cascade must be carefully regulated to achieve a protective response without causing adverse impact on the host. Studies in malaria-endemic regions have found that it is important to have a balance between a host pro-inflammatory, Th1 response (e.g., TNF-alpha, IL-6, IL-12, and interferon-gamma) and anti-inflammatory, Th2 response (IL-4, IL-10, and others)^29,30^; severe manifestations of malaria are often due to excessive pro-inflammatory responses. The same appears to be true in at least some cases of COVID-19,^18^ suggesting that a coinfection that also leads to excess pro-inflammatory responses might result in more severe manifestations and poor prognosis.

Malaria-induced immunosuppression has also been observed in many coinfections, significantly inhibiting immune responses to the other infection (e.g., to *Salmonella* spp.).^31,32^ However, malaria-induced immunomodulation has been shown to be protective against severe manifestations of some respiratory viruses. In Kenya, hospitalized children diagnosed with influenza and malaria were less likely to experience respiratory distress than those with influenza alone.^33^ Coinfection with *Plasmodium* spp. could suppress the production of pulmonary cytokines and decrease the recruitment of cellular inflammatory components to the lungs, leading to reduced clinical symptoms and inflammation, as was found during pneumovirus infections in a murine model. However, in the murine model, viral control was also impaired, leading to increased viral dissemination.^34^ Similar dynamics could occur during *Plasmodium–*SARS-CoV-2 coinfection; malaria-induced immunosuppression might lead to milder manifestations of COVID-19 but simultaneously decrease viral control, potentially increasing or sustaining viral loads, which could increase the potential for viral transmission.

### Age-related vulnerability to malaria and COVID-19.

Susceptibility to malaria in highly endemic areas differs by age: younger children are more vulnerable to malaria infections and at a higher risk for severe malaria.^13^ For COVID-19, children are less likely to develop severe disease, whereas older populations are disproportionately affected, with a higher risk of severe disease and death.^35^ This may be due to the fact that children are more likely to produce T-regulatory cytokines (IL-10, IL-23, and IL-6) and have less inflammation (because of their immature immune systems) than older people who mount a more pro-inflammatory cytokine cascade, potentially contributing to pathogenesis.^35^ How age-related susceptibility to COVID-19 will play out in Africa, where many children are immunologically stimulated by several infections in addition to malaria, is not clear. Importantly, malaria infections in endemic areas frequently result in chronic, afebrile disease in older children and adults.^36^ It remains unknown whether this underlying infection will alter susceptibility to or severity of COVID-19 in these populations; it is important that surveillance systems be modified to collect data to inform our understanding of this issue.

### Respiratory distress and ARDS.

Respiratory distress, observed in up to 25% of adults and 40% of children with severe *P. falciparum* malaria, has several causes, including severe anemia, metabolic acidosis, cytoadherence of infected erythrocytes in pulmonary vasculature, and coinfections with pneumonia-causing pathogens.^37^ The clinical spectrum varies from mild upper respiratory symptoms to acute lung injury and fatal ARDS. Acute respiratory distress syndrome is rare in young children with malaria but occurs in 5–25% of adults and 29% of pregnant women with severe *P. falciparum* infections, and less commonly with *P. vivax* malaria.^37^ In both malaria and COVID-19, ARDS is linked to inflammatory cytokine–mediated increased capillary permeability or endothelial damage, which results in major alveolar damage.^38–40^ Given this situation, *Plasmodium* spp.–SARS-CoV-2 coinfections may result in particularly rapid deterioration, with a poor prognosis. As the inflammatory-mediated alveolar damage in malaria-induced ARDS progresses even after treatment and parasite clearance,^37^ coinfected individuals may be prone to severe COVID-19. Because both malaria and COVID-19 can lead to similar clinical manifestations, including fever and respiratory symptoms, one or the other may be overlooked in a differential diagnosis of respiratory distress, leading to increased fatalities. As SARS-CoV-2 transmission increases in LMICs, particularly in Africa and India, clinicians should keep this in mind. In addition, documenting the frequency, distribution, and outcomes of these coinfections is important.

### Anemia.

Anemia is highly prevalent in LMICs and results from multiple causes. In cross-sectional household surveys in sub-Saharan Africa, 61%, 33%, and 3% of children younger than 5 years had any anemia, moderate anemia, and severe anemia, respectively.^13^ More than one-fifth of children with malaria develop SMA, with a CFR of 8.4%.^41^ Whereas the hematologic sequelae of COVID-19 are still being elucidated, a meta-analysis describing 1,210 COVID-19 patients from four studies found that hemoglobin values were 0.71 g/dL (95% CI: 0.59–0.83 g/dL) lower in individuals with severe disease versus milder disease.^42^ Whether lower hemoglobin is a risk factor or a sequela of severe COVID-19 disease is unknown. However, because of limited reserves, even small perturbations in oxygen-carrying capacity in individuals with preexisting malarial anemia may result in insufficient tissue oxygenation in the midst of COVID-19–induced respiratory failure.

### Pro-coagulant state.

Numerous viral infections, including SARS-CoV-2, induce a pro-coagulant state through the induction of tissue factor expression, endothelial dysfunction, von Willebrand factor elevation, and Toll-like receptor activation.^43,44^ Markers of a hypercoagulable state, including increased D-dimer and fibrin degradation product levels, and prolonged prothrombin time are associated with a poor prognosis.^45^ Clinically, the hypercoagulable state manifests with a high rate of venous thromboembolism and arterial thrombotic complications (including pulmonary embolism and stroke).^46,47^ COVID-19 patients are at risk for developing disseminated intravascular coagulation (DIC),^45,48^ and autopsy findings have included both pulmonary hemorrhage and thrombosis.^49^ Thrombocytopenia is another potential feature of COVID-19, thought to be due to excessive activation of the coagulation cascade, leading to platelet activation and subsequent consumption,^44^ and is associated with worse outcomes.^50^

Malaria is also associated with a pro-coagulant state, with activation of the coagulation cascade, mediated by TNF-alpha and IL-6, proportional to disease severity.^51^ Whereas micro-thrombotic complications are most commonly described, thrombosis of large vessels, including cerebral venous thrombosis, and pulmonary embolism have been described.^52,53^ Thrombocytopenia develops in 60–80% of malaria cases.^51^ Although bleeding and DIC are rarely seen, occurring only in severe malarial cases accompanied by coagulopathy,^54^ they are associated with high mortality.^51^ Lysis of activated platelets, along with tissue factor released from damaged vascular endothelial cells, promotes the pro-coagulant state,^54^ similar to the proposed mechanism in COVID-19. Thus, *Plasmodium* spp.–SARS-CoV-2 coinfection could lead to even greater degrees of coagulopathy and more severe disease than with either infection alone.

### Potential interactions between NTDs and COVID-19.

Helminths, including stool-transmitted helminths (STH), schistosomes, and filariae, typically push the immune system toward anti-inflammatory Th2 pathways through a variety of regulatory mechanisms.^55,56^ Protozoal parasites, such as trypanosomes or *Leishmania* spp., are more likely to induce a Th1, pro-inflammatory response. However, there are many deviations from this characterization. Some helminths induce Th1 responses in some stages of the life cycle (e.g., microfilariae of filarial parasites or schistosome eggs), resulting in symptomatic disease, but Th2 responses in other stages (e.g., adults of both filarial parasites and schistosomes). The downregulation of the inflammatory response associated with helminths may reduce the development of immunity or response to vaccines, decrease inflammation associated with autoimmune diseases, reduce the ability to control *Mycobacterium tuberculosis* and *Mycobacterium leprae* coinfections, and reduce the severity of malarial coinfection. The pro-inflammatory effects of some protozoal infections may worsen the severity of some, but not all, viral infections.^55,57^ In addition, polyparasitism is quite common, and the overall impact on inflammation depends on the sequence of infections and burden of each.^58^ Thus, coinfection with parasitic NTDs could result in altered risks and severity of clinical manifestations of SARS-CoV-2 infection, with the potential for decreased development of immunity with increased viral loads.

The severity of COVID-19 has been associated with underlying health conditions that usually occur with advancing age. Several NTDs, if left untreated, can result in chronic sequelae in much younger populations. For example, because acute *Trypanosoma cruzi* infection is typically asymptomatic or results in a mild, self-limited illness, it is frequently undetected and left untreated. Yet, in young or middle adulthood, 20–30% of persons chronically infected with *T. cruzi* develop cardiac manifestations, commonly a complex, dilated cardiomyopathy.^59^ For these individuals, coinfection with SARS-CoV-2 could be life-threatening. STH infections may result in anemia^60^; if, as described previously, anemia predisposes individuals to more severe outcomes, then coinfection of STHs and SARS-CoV-2 in children and pregnant women could be problematic.

### Malnutrition and COVID-19.

Chronic malnutrition is associated with both malaria^61^ and NTDs,^62^ and is relatively common among children in sub-Saharan Africa as well as parts of Latin America and Asia. Prealbumin, a marker for protein malnutrition,^63^ was found to be lower on admission in patients with COVID-19 who developed ARDS than on those who did not.^64^ Although lower prealbumin may be a marker for more severe disease, immunosuppression associated with undernutrition preceding infection with SARS-CoV-2 could exacerbate the severity of COVID-19.^65,66^ Undernutrition is thought to have led to excess mortality with both the 1918 and H1N1 influenza pandemics.^67,68^ Given relatively high rates of undernutrition among children in LMICs (12.3%),^69^ an association between undernutrition and clinical severity of COVID-19 could increase the proportion of severe illness above current predictions, particularly among children.

## CONCLUSION

Although SARS-CoV-2 has spread globally, our understanding of the epidemiology and clinical course of COVID-19 in countries with substantial burdens of malaria and NTDs is just beginning, in part because community transmission generally started later in these countries and because testing for SARS-CoV-2 is limited in most LMICs. Although current predictive models suggest lower mortality rates in LMICs than in high-income countries, if coinfections with malaria or parasitic NTDs increase complications with SARS-CoV-2 infections and there is a shift in the age pattern of comorbidities to younger ages, then the burden of COVID-19 in LMICs may be substantially worse than predicted, and potentially higher than the burden in high-income countries.^70^ If a shift to a Th2 response is more common, and if that shift provides some protection from severe disease while reducing long-term immunity or increasing the time frame of viral shedding, the epidemiology of COVID-19 in LMICs could be substantially different from what has been seen elsewhere.

Rapidly developing surveillance platforms to monitor signals of SARS-CoV-2 coinfection with malaria or other NTDs will be critical. One early indication of a potential interaction would be a shift in the age pattern of severe COVID-19, with higher rates of clinical disease in children than has been observed in China, Europe, or North America. However, more definitive information on coinfections and outcomes will be needed to interpret such shifts. Efforts to characterize COVID-19 cases in LMICs, such as the WHO First Few X cases protocol,^71^ and addition of SARS-CoV-2 testing to influenza sentinel surveillance^72^ could be expanded, based on local prevalence of malaria and NTDs, to include testing for malaria and NTDs. Such additional testing could help determine rates of coinfection and compare severity of outcomes by infection status. Additional efforts to more carefully describe the clinical impacts of coinfections can follow. These efforts are important to understanding the potential impact of COVID-19 on LMICs and for mitigating against the worst outcomes.
